# LSTM-based recurrent neural network provides effective short term flu forecasting

**DOI:** 10.1186/s12889-023-16720-6

**Published:** 2023-09-14

**Authors:** Alfred B. Amendolara, David Sant, Horacio G. Rotstein, Eric Fortune

**Affiliations:** 1 Department of Biomedical Science, Noorda College of Osteopathic Medicine, Provo, USA; 2 Federated Department of Biology, New Jersey Institute of Technology, Newark, USA

**Keywords:** Influenza, Modeling, Machine learning, LSTM, Epidemiology

## Abstract

**Background:**

Influenza virus is responsible for a yearly epidemic in much of the world. To better predict short-term, seasonal variations in flu infection rates and possible mechanisms of yearly infection variation, we trained a Long Short-Term Memory (LSTM)-based deep neural network on historical Influenza-Like-Illness (ILI), climate, and population data.

**Methods:**

Data were collected from the Centers for Disease Control and Prevention (CDC), the National Center for Environmental Information (NCEI), and the United States Census Bureau. The model was initially built in Python using the Keras API and tuned manually. We explored the roles of temperature, precipitation, local wind speed, population size, vaccination rate, and vaccination efficacy. The model was validated using K-fold cross validation as well as forward chaining cross validation and compared to several standard algorithms. Finally, simulation data was generated in R and used for further exploration of the model.

**Results:**

We found that temperature is the strongest predictor of ILI rates, but also found that precipitation increased the predictive power of the network. Additionally, the proposed model achieved a +1 week prediction mean absolute error (MAE) of 0.1973. This is less than half of the MAE achieved by the next best performing algorithm. Additionally, the model accurately predicted simulation data. To test the role of temperature in the network, we phase-shifted temperature in time and found a predictable reduction in prediction accuracy.

**Conclusions:**

The results of this study suggest that short term flu forecasting may be effectively accomplished using architectures traditionally reserved for time series analysis. The proposed LSTM-based model was able to outperform comparison models at the +1 week time point. Additionally, this model provided insight into the week-to-week effects of climatic and biotic factors and revealed potential patterns in data series. Specifically, we found that temperature is the strongest predictor of seasonal flu infection rates. This information may prove to be especially important for flu forecasting given the uncertain long-term impact of the SARS-CoV-2 pandemic on seasonal influenza.

## Introduction

Influenza virus is responsible for a recurrent, yearly epidemic in most temperate regions of the world. According to the CDC, in the 2017-2018 season alone, influenza virus was responsible for 79,000 deaths and nearly 1 million hospitalizations [1]. Since the emergence of the SARS-CoV-2 virus, flu-like illness has dropped, however it still presents a remarkable burden on the medical system. For the 2021-2022 season the CDC reports 5,000 deaths and 100,000 hospitalizations, a significant number despite the confounding presence of SARS-CoV-2 [2]. Seasonal variance in flu burden, while well established, is not well understood [3]. Modeling can provide a means to better understand seasonal flu patterns, as well as provide a practical tool for public health officials. In order to model influenza effectively it is important to explore the genetic variability of influenza as well as the various climate and population factors that may contribute to seasonality.

Unfortunately, recombination and re-assortment can result in rapid and extreme antigenic shifts in the influenza virus. This presents a challenge to modeling flu trends as strains may vary considerably from one year to the next, which is highlighted by the 2009 Swine Flu pandemic, whose titular strain was likely a result of a single amino acid substitution in the protein PB1-F2 [4].

In addition to genetic variability, the mechanism of influenza transmission is of great importance to modeling and presents even more challenges. There are three main ways by which influenza virus may be transmitted. One, direct contact between an infected individual and a non-infected individual or secondary contact via some surface such as a door knob [5]. Two, large droplets expelled by an infected person while coughing or talking may distribute viral particles up to 1m [5, 6]. Three, small aerosol droplets, generally defined as $<5\mu$m, may be expelled by infected patients [5, 7, 8]. This final form of transmission is likely the primary source of infection as small particles remain airborne for the longest time and are able to reach the lower respiratory tract most easily [5]. Once a patient is infected with influenza, some time may pass prior to displaying symptoms [9, 10]. This is an important consideration when observing transmission and incidence rates, as a patient may spread the virus prior to displaying clinical symptoms and may delay seeing a doctor for several days after infection.

Given the various modes of transmission as well as the expected delay in identification of an infected individual, it is reasonable to infer that increased proximity of infected and susceptible individuals indoors during the winter months is one driver of seasonal spikes in influenza incidence [11]. However, there is no firm consensus on what causes the seasonal variability, but temperature, dry air, and host immune irregularities may play a role [3, 12, 13]. Additionally, despite prior exposure, novel viruses emerge that can evade host immune responses. This further increases yearly variability [13]. Ultimately, yearly variability may be due to very small changes in a multitude of variables that are amplified by population dynamics [14]. Interestingly, tropical regions do not show strong seasonality. Instead they have generally flat ILI incidence that varies with the rainy season [3, 15]. These minute changes, and seemingly contradictory patterns, create a complex and difficult to model phenomenon that requires a unique approach.

Traditional deterministic approaches to modeling may struggle to integrate this myriad of factors. Given the naturally time-dependant nature of influenza rates, a potential solution is the use of LSTM (Long-Short-Term-Memory) nodes in a neural network [16, 17]. Neural networks are complex models containing interconnected discrete algorithms called nodes. LSTM nodes were designed to solve disappearing or exploding gradients, a common problem in recurrent neural networks [18]. Gradients are an integral part of neural networks, they affect the “on/off” signals of the individual nodes. Depending on the data set and hyper-parameters of the model, gradients can run out of bounds. LSTM nodes circumvent this problem by introducing a CEC or constant error carousel [19]. The CEC allows for gradients to remain unchanged from one node to the next. The more recent addition of a “forget gate” allows the LSTM node to reset, further reducing gradient runaway [18]. LSTM based neural networks allow for complex time-series forecasts. They are an ideal candidate for influenza prediction and provide a relatively novel foundation for forecasting. This technique, when applied to influenza, performed better than random forest regression, support vector machines, and ARIMA (auto-regressive integrated moving average) in previous literature [20].

It is the goal of this paper to develop robust model architecture using an LSTM based neural network to provide the basis for practical forecasting as well as insight into the various features that may impact seasonal influenza trends.

## Methods

In the following two subsections, the data acquisition and the model building processes are detailed. Supplemental information on the data sets and code related to the model may be found online [21]. Data were processed in R (version 4.2.0) and Python (version 3.9.13). Final data manipulation was done using Python. The models were designed and constructed in Python using TensorFlow 2.1 and the Keras API running natively on Windows 10. LSTM nodes were created via standard Keras implementation. TensorFlow used GPU acceleration. The computer used to train and run the model has the following specifications: AMD Ryzen 5 5600X @ 4.65GHz, 32GB DDR4 RAM, RTX 3060 Ti 8GB.

### Data compilation

**Fig. 1:**
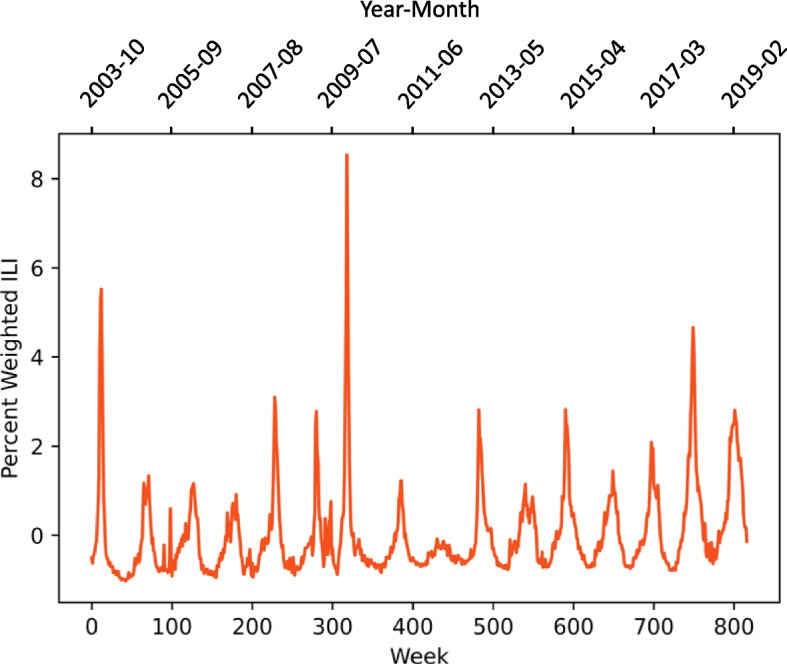
Weekly ILI from 2003 to 2019 reveals regular, repeating outbreaks. Peak influenza incidence occurs each year during winter months. The exception is the 2009 flu season, now known as the Swine Flu pandemic, which can be found centered at approximately week 300. This pandemic season was unusual in that ILI incidence remained elevated through the spring and summer

CDC Region 1 (New England), containing Connecticut, Maine, Massachusetts, New Hampshire, Rhode Island, and Vermont, was selected as the target region due to strong seasonality. The initial data set from the CDC Flu View was downloaded as a Comma-Separated-Values (CSV) file. This set contained ILI percentages, total patients, and information on strain subtype. Influenza-like-illness percentage is the percent of outpatient visits that are due to flu-like symptoms without another confirmed diagnosis [22]. Data ranged from week 40 of the 2003-2004 flu season to mid 2018-2019 flu season (Fig. 1). The data were trimmed to include up to week 21 of the 2018-2019 season. This range, from the week of 5 October 2003 to the week of 26 May 2019, was used for all other data collected.

In addition to the raw data, the CDC calculates a regional baseline for each year, which was included in this data set. A regional baseline was available from the 2007-2008 season onward. In order to fill in missing baselines for the previous several seasons (from the 2003-2004 season to the 2006-2007 season), the CDC procedure was followed as closely as possible. Beginning with the 2003-2004 season, a 1-year baseline was calculated since years prior to 2003 did not report off-season ILI levels. The next year had a 2-year baseline, and the following year onward had a 3-year calculated baseline until the reported 3-year baseline was available. The estimated baselines were adequate for the purposes of this model. All data were reported as weekly incidence. A total of 816 weeks were included.

Climate data were taken from the National Oceanic and Atmospheric Administration’s Climate Data Online [23]. In order to provide a sample representative of the region, a single monitoring station was selected from each state for a total of 6 weather stations. These stations include Hartford Bradley Airport, Connecticut; Boston, Massachusetts; Augusta Airport, Maine; Mt. Washington, New Hampshire; Providence, Rhode Island; Montpelier, Vermont. Most available data were reported as daily averages, with a small portion of the temperatures reported as monthly averages. All data were converted to weekly data and trimmed to match the collected CDC data. The mean of all stations was then calculated to produce weekly aggregate data, which were included in the final data set. The climate factors used were average temperature, average wind speed, and precipitation.

In addition to climate factors, time spent indoors was identified as a potential predictor. Heating and cooling degree days are used to estimate the amount of heating and cooling costs but can also be used as a proxy marker for the amount of time individuals spend indoors. Heating and cooling degree days are calculated as the difference between a day’s average temperature and $65^{\circ }$F [24]. This data was also accessed from the National Oceanic and Atmospheric Administration’s Climate Data Online [23].

Population data were taken from the U.S. Census Bureau. These data included population totals and immigration data from each state in the CDC New England region [25].

Finally, vaccination data and vaccine estimated effectiveness were obtained on a regional basis from the CDC [22].

The final data set was limited to the CDC-defined New England region. All data was converted to weekly averages. Data were available for the entirety of the timeframe selected, with the exception of wind speed in Rhode Island, which was estimated using the average reported wind speeds from the previous decade.

### Building and training the model

**Fig. 2:**
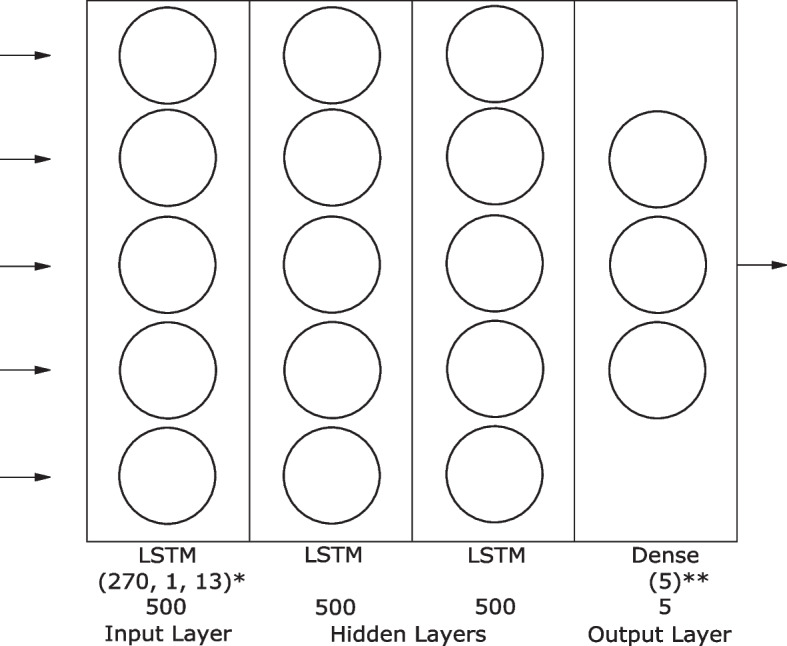
Final model architecture. The final model contained 4 layers total. An initial 500 node LSTM input layer with a variable shape, 2 hidden LSTM layers with 500 nodes each and a dense output layer with a variable output shape. *input shape varies with data shape **output shape varies with label shape

The model presented in this paper was a recursive deep neural network made up of a bidirectional LSTM input layer, two bidirectional LSTM hidden layers and a dense output layer with variable output nodes (Fig. 2). The basic structure of an LSTM as implemented in Keras includes a forget gate, an input gate, and an output gate. This model was based on a model previously proposed architecture [26].

Data were initially reshaped into a 3-dimensional array and then broken into time-steps that represented one week’s data. Prior to reshaping, data were standardized using the following z-score normalization equation :

1 $$\begin{aligned} \frac{(x - mean)}{standard\ deviation} \end{aligned}$$

Once the data were reshaped and standardized, they were broken into training and testing sets. In order to make the best use of limited data, several configurations were used. Initial training and validation involved breaking the available data into equal series of data which varied to assess generalizability and to investigate underlying patterns in the data.

Parameters were assessed individually, and the model was tuned incrementally. Data variables were removed systematically to determine impact on predictive performance. Mean square error, mean absolute error, and R-squared values were used as metrics to determine model accuracy and control learning. Additionally, a recursive function was added to improve long term prediction out to 10 weeks. Finally, future climate data, for example temperature +1 week, was added in order to simulate adding weather forecasting data. Further hyper-parameter tuning was performed to achieve best performance. Validation of the optimized model was accomplished using k-fold cross validation with k=3 as well as forward chaining cross-validation with k = 10. The model was further evaluated by comparing performance to multiple linear regression (MLR), k-nearest neighbor, gradient boosting (GBT), extreme gradient boosting (XGBoost), and multi-layer perceptron (MLP). Models were chosen to represent widely available, easily implemented algorithms that might be applied to this problem. We have excluded a basic recurrent neural network (RNN) as we feel that LSTM-based RNNs are a direct evolution of simple RNNs and therefore not an inherently different class of model. These additional models were implemented and validated using SciKit-Learn and Scalecast with two training splits, 80%/20% and 67%/33%.

Select hyper-parameters for the model are listed below:

- Max Epochs = 500
- Batch Size = 270
- Validation Split = 0.2
- Minimum Learning Rate = 0.0001
- Time-Lag = 4 weeks

### Simulated data production

Simulation data was produced in order to verify the impact of temperature on prediction accuracy, and also to provide further validation of predictions. Influenza infection data was generated using a modified SIRS model incorporating yearly antigenic drift. Temperature and precipitation data was approximated by generating sine waves with Gaussian noise. The tuned final model, trained on the entirety of the real data set, was then used to generate predictions on the simulated data set.

## Results

### Training on two-thirds of the data set provides the adequate performance

Using the complete data set and a time lag of one week, nine different training sets were used to train models. These training sets were divided into three groups of 400-week training sets, 540-week training sets and 700-week training sets. MAE (mean absolute error) was used to determine relative performance along with visual interpretation of predictions. MAE was recorded for weeks +1, +5 and +10 (Table 1). The best performance was achieved when predicting one week in advance. Both MAE and the standard deviation of the error rose substantially by week +10. Two sample t-tests were used to determine significant differences between week 1 predictions from each training set. There was significant difference between different frame shifts within all three training-set-length groups. The mean increase in MAE from week 1 to week 10 was 0.1661. There was no significant difference between the 540 and 700 week training sets, although the 400 week training set performed significantly worse. Moving forward, 540 week training sets were used for testing as they provided sufficient predictive ability and were easier to manipulate.

**Table 1:** A training-testing split of 540-270 provides the best predictions in a baseline model. Bolded text indicates week averages

	*Training Set*	*Prediction Error (MAE)*		
		*Week 1*	*Week 5*	*Week 10*
*400 Weeks*	Weeks 100 - 500	0.6630	0.6637	0.6664
	Weeks 200 - 600	0.5370	0.5274	0.5394
	Weeks 300 - 700	0.3374	0.6682	0.6440
	**Average**	**0.5124**	**0.6197**	**0.6166**
*540 Weeks*	Weeks 0 - 540	0.3103	0.4678	0.5792
	Weeks 0 - 270 & 540 - 806	0.3860	0.5399	0.5563
	Weeks 270 - 806	0.3130	0.3609	0.3878
	**Average**	**0.3364**	**0.4562**	**0.5077**
*700 Weeks*	Weeks 0 -700	0.5227	0.5806	0.7309
	Weeks 53 - 753	0.3086	0.6008	0.6094
	Weeks 106 - 806	0.4263	0.6601	0.5861
	**Average**	**0.4192**	**0.6138**	**0.6421**

### Temperature and precipitation are the strongest predictors of ILI

Using the 540:270 training:testing data split determined above, individual variables were systematically removed. The data set became progressively smaller until only data columns ‘percent ILI’, ‘Week’, and ‘Year’ remained. Temperature was the most important variable for predicting week +1. Precipitation also had a significant effect when removed. Removing either of these variables reduced performance of week +1 predictions. Removing population and vaccination data appears to have improved predictive power substantially. Removing monthly temperature and precipitation, weekly precipitation, and weekly temperature decreased predictive performance. The best predictions were obtained with a data set containing only precipitation and average temperature (Table 2).

**Table 2:** Temperature is the strongest climate predictor of ILI. Base model here includes all data. Each MAE indicates model performance with the sequential removal of the listed parameter and all prior parameters

*Parameter*	*Prediction Error (MAE)*
**Base Model**	**0.213**
Average Wind Speed - Monthly	0.182
Precipitation - Monthly	0.218
Average Temperature - Monthly	0.204
Population	0.195
Vaccine Effectiveness	0.195
Vaccination Rate	0.187
Average Wind Speed - Weekly	0.163
Precipitation - Weekly	0.185
Average Temperature - Weekly	0.231

Bolded text indicates baseline performance

### Four week time lag provides optimal performance

A model using the reduced data set, a 540:270 split, and a time lag of one week served as a baseline for evaluation. Time lags of -4, -12, -16 and -52 weeks were compared to this baseline. A time lag of -4 weeks provided an average decrease of 0.1400 percent-ILI error across weeks +1, +5 and +10. The greatest improvement was seen in week 10 predictions. Predictive performance degraded as the time lag increased past 4 weeks (Table 3). This preference for a 540-week training set may be related to the underlying structure of the model, which contains 500 nodes per hidden layer.

**Table 3:** A 4 week time lag provides the best predictive performance vs. baseline

	*Prediction Error (MAE)*		
*Time Lag*	*Week 1*	*Week 5*	*Week 10*
**Baseline**	**0.3412**	**0.4485**	**0.4963**
t-4	0.2903	0.2882	0.2876
t-12	0.4000	0.4867	0.4709
t-16	0.3831	0.4592	0.4496
t-52	0.4838	0.5127	0.4868

Bolded text indicates baseline performance

### Validation reveals potential pitfalls in the data processing

A series of validations was performed on the final model derived from the above tuning. During 3-fold cross validation, the model achieved an average MAE of 0.210501 at week +1, 0.383306 at week +5, and 0.378014 at week +10. During forward chaining cross validation however, the average MAE’s of weeks +1, +5, and +10 were 0.228381, 0.473647, and 0.716970 respectively. While week +1 predictions remained consistent, variation in the training and testing sets impacted the model substantially. Notably, the validation chain revealed several problem slices identified at weeks k = 3, 6, and 9. Poor performance at these sections resulted in elevated average MAE as well as higher than expected standard deviation of the MAE at both weeks +5 and +10 (week +5 = 0.116900, week +10 = 0.568778). Slice k = 3 was discovered to contain the 2009 Swine Flu pandemic, which the model struggled to accurately predict due to the magnitude of the data. Slice k = 5 likely resulted in poor performance as the initial values at time 0 were exceptionally high resulting in inflated week +5 and week +10 errors. Slice k = 10 showed consistent under prediction. From this we can gather that the model is sensitive to the time span of training and testing data, as well as outlying features that may be present, such as the comparatively huge spike of the 2009 pandemic.

### Prediction of simulation data provides insight into model parameters

**Fig. 3:**
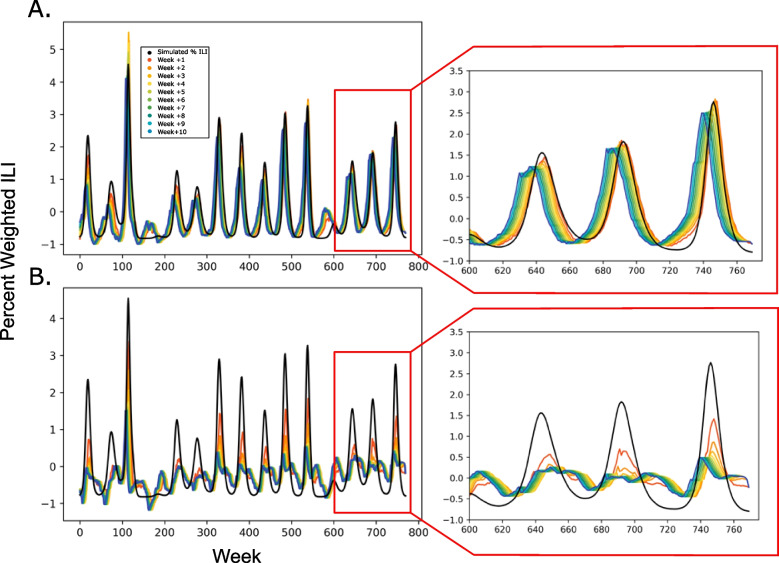
The proposed model is able to predict simulation data when trained on real data. **A** Predictions from +1 to +10 weeks made on simulated data including temperature and precipitation. **B** When temperature data is shifted out of phase, the model is unable to predict trends in ILI rates. Inserts present a zoomed in view of the final 175 weeks, for easier visual assessment of prediction accuracy

A model trained with the optimized data set and a 4-week time lag was used to predict fifteen years of simulated ILI data generated by a modified SIRS model. The LSTM-based model was able to achieve a MAE of 0.1827 at week +1, 0.3233 at week +5, and 0.3242 at week +10. When temperature data was shifted out of phase, prediction accuracy dropped at all three time points to 0.4731, 0.7069, and 0.7149 respectively (Fig. 3). This is a strong indicator that not only is temperature important for predictive accuracy, but that it may be integral to the generation of the cyclical infection rates seen with influenza.

### LSTM based model outperforms comparison regression models

**Fig. 4:**
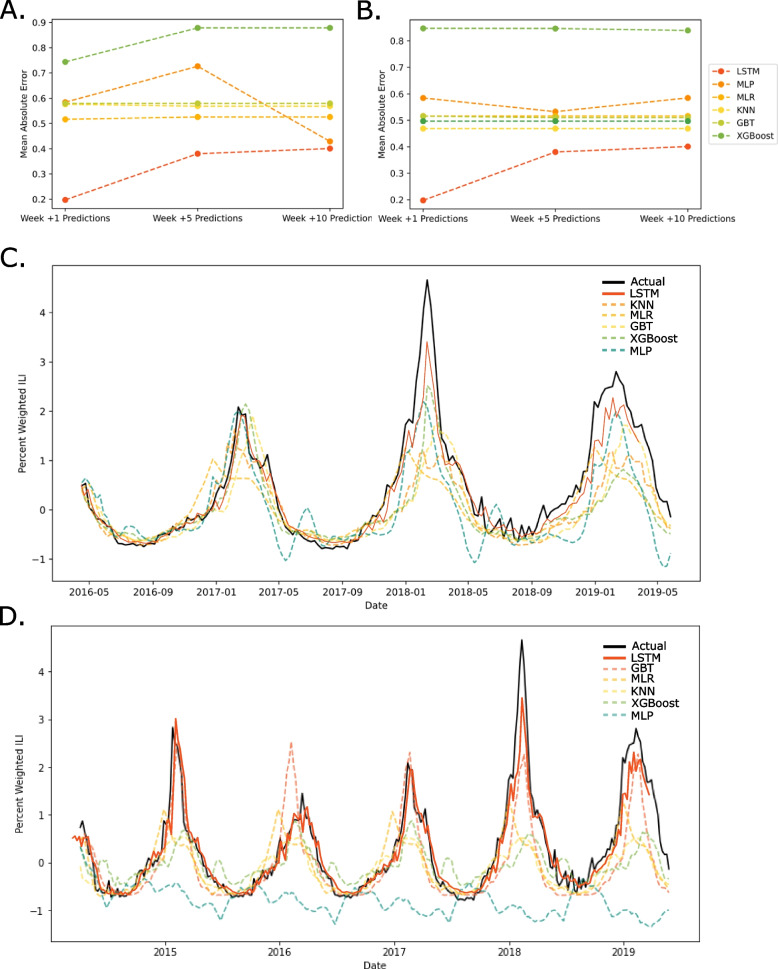
The final LSTM model outperformed 6 other comparison models at +1, +5, and +10 weeks. **A** The LSTM model achieves a lower MAE in all weeks compared to models trained on a uni-variate data set. **B** The LSTM model achieves a lower MAE in all weeks compared to models trained on a multivariate data set including precipitation and temperature data. **C** +1 week predictions trained on 80% of the data set. The best performing comparison model at +1 weeks was KNN. LSTM model predictions and actual data have been superimposed for comparison. **D** +1 week predictions trained on 67% of the data set. The best performing comparison model at +1 weeks was GBT. LSTM model predictions and actual data have been superimposed for comparison

The LSTM-based model achieved lower MAE at +1, +5 and +10 weeks than any other model (Fig. 4 A & B). All models except multi-layer perceptron and extreme-gradient boosting improved when applied to the multivariate data set including percent ILI, average temperature, and precipitation. The comparison model with the next best performance, after LSTM, was k-nearest neighbor, although multi-layer perceptron performed nearly the same as LSTM at +10 week when trained on uni-variate data. The performance of multi-layer perceptron and K-nearest neighbor degraded when trained on a smaller set, while gradient boosting improved to achieve the second-best performance model after LSTM. Visual assessment of LSTM models versus comparison models trained on both 66% and 80% of available data also favor LSTM performance at +1 week (Fig. 4 C & D). Overall, comparison models were not able to match the proposed LSTM based model’s ability to predict granular changes or larger trends.

## Discussion

Influenza produces seasonal outbreaks that have large economic and human costs. Currently, our best defense against seasonal outbreaks is widespread vaccination. However, despite advances in virology, epidemiology, and immunology, an influenza vaccine that produces long-lasting immunity has eluded researchers, and annual vaccines have effectiveness as low as 20% [27, 28]. Additionally, major pandemic seasons can occur unexpectedly. As a result, predicting when and how any given flu season progresses is of great importance.

### Data and training trends

Of the climate variables, temperature was the strongest outcome predictor. This was supported both by the initial parameter testing and the simulated data prediction. When temperature data was shifted out of phase with ILI, it significantly reduced predictive accuracy. This is in agreement with prior literature describing seasonal flu patterns. Precipitation was a second strong predictor, possibly as a result of precipitation’s correlation with humidity. Although the actual effect of relative humidity on influenza virus transmission has been contested, it has been shown to be useful as a predictor [15, 29, 30]. The decrease in error due to addition of precipitation suggests that it may be used in place of humidity data, which is often sparse and less uniform.

Notably, adding future climate data to the model greatly improves predictive performance, further indicating that select climate data may be a useful predictor of ILI trends. Unfortunately, quality climate data can be difficult to gather. In this case, data was averaged to create regional approximations. It is possible that if the model was applied to a smaller geographical area with more uniform weather, the predictive effect of climate data may be even greater. More granular data seemed to have a larger effect than less granular data. Weekly averages derived from daily data had a larger impact than weekly averages derived from monthly data.

The optimal training split for this model and data set was found to be 540:270. This is likely related to the structure of the input nodes. While there is no commonly accepted method for determining optimal layer size, there exist a variety of methods to estimate optimal node numbers and all agree that there is a relationship between input layer size, problem complexity, and node number [31].

### Limitations

Data used to train this model was limited to publicly available repositories. Additionally, data across organizations was not formatted comparably, necessitating considerable restructuring. Higher quality, more consistent data could provide a measurable improvement to model performance. In particular, climate data varied considerably. Potentially using more stations, or better selected stations may provide better correlated data. Investigating areas with more uniform weather could also reveal more robust patterns. This report focuses on a larger geographic region due to the formatting of CDC data and limited compatibility with climate data.

Interestingly, removing population and vaccination data had no effect and increased performance, respectively. While it is possible that these factors are not useful predictors, it is more likely that the data available was not adequate to reveal underlying patterns. The population data used in this study was limited to regional total populations and the vaccination data was limited to national data. If more specific, granular data could be collected, it may prove a useful variable in predicting influenza. Notably, yearly vaccine effectiveness can only be calculated retrospectively, limiting its use as a potential predictor. Despite the lack of impact of population data, it would likely be valuable if a spatial dimension were included in the model.

In addition to data structure limitations, the application of LSTM nodes with their dependence on time series relationships, limits the options for robust validation, especially in the setting of limited flu data. Data cannot be randomly shuffled and often n-fold cross validation presents misleading results due to the nature of the time-series. Additionally, comparison models were generated and trained automatically via python packages, and the automatic tuning of hyper-parameters may have widened the gap in performance.

### Practical applications and future directions

Using a variety of techniques, including recursive predictions, models can be stretched to predict to an indefinite point in the future. However, predictive performance for the model presented plateaus at about 10 weeks. Good one-week predictions show that this approach is practical for now-casting, which would allow for prediction of rates the following week.

The primary advantage of this model is the straightforward architecture. It is small and does not require a significant amount of computational power while retaining better performance than alternative methods of regression. Once the model has been designed and implemented, new data can be continuously added. A simple pre-processing pipeline could allow for seamless addition of real-time climate and influenza data to the model, allowing for automatically updated predictions. This model also provides a framework for future research as training and prediction time is short, allowing rapid testing and impromptu modifications.

Outside of potential practical applications, the proposed model may expand our understanding of real-world flu dynamics. Our findings strongly suggest that temperature and precipitation play a significant role as seasonal drivers of ILI, reinforcing existing knowledge in this area. It is important to emphasize that our proposed model exhibits sensitivity to changes in these parameters, indicating its ability to detect relationships within the data. Although our results show that predictive accuracy is not heavily influenced by other parameters, it does not imply that these factors do not impact flu dynamics in the real world. To further elucidate underlying patterns, especially patterns that were not evident here, this model should be applied to various regions, particularly those characterized by variable climate factors. We believe that applying LSTM to data from other regions will allow for discovery of the factors that determine seasonal ILI rates. By including known drivers, as well as incorporating additional climate and population data, we aim to draw meaningful conclusions about the underlying phenomenon. However, it is important to note that this endeavor falls outside the scope of the current paper and will be explored in future research.

## Conclusions

Predictions were made for tests sets of various lengths and frames. Baseline performance was determined, then the most effective time lag was selected, and finally, the data set was evaluated. Overall performance for each model was established using MAE, ME (mean error), standard deviation, and visual analysis. Week 1 predictions were the most accurate. Predicting further than 5 weeks was influenced heavily by time lag, modeling method, and data selection. The most significant increases in performance were achieved by tuning the time lag and by using the recursive prediction function.

Overall, the effectiveness of LSTM-based models as a predictive tool is supported by the results presented here. While machine learning may act as a “black box” with opaque inner workings, continuous application to a biological question may provide a useful practical tool as well as reveal previously unknown patterns in a system. Given its effectiveness compared to other regression methods, this model could be rapidly applied to nearly any infectious disease that acts in a time-dependent fashion.

## Data Availability

The data and code that support the findings of this study are openly available on GitHub at https://github.com/aamendolara/flu-modeling.git. These data were derived from the following resources available in the public domain: 1 CDC data: https://gis.cdc.gov/grasp/fluview/fluportaldashboard.html 2 National Oceanic and Atmospheric Administration climate data: https://www.ncdc.noaa.gov/data-access/land-based-station-data/land-based-datasets 3 US Census population data: https://www.census.gov/data.html

## Abbreviations

ARIMA: Auto-regressive integrated moving average
CDC: Centers for Disease Control and Prevention
CSV: Comma Separated Value
ILI: Influenza-Like-Illness
GBT: Gradient Boosting
XGBoost: Extreme Gradient Boosting
KNN: K-Nearest Neighbor
LSTM: Long Short Term Memory
MAE: Mean Absolute Error
ME: Mean Error
MLR: Multiple Linear Regression
MLP: Multi-Layer Perceptron
NCEI: National Center for Environmental Information
